# The association of miR‐27a rs895819 polymorphism with colorectal cancer risk in Chinese population

**DOI:** 10.1002/jcla.23497

**Published:** 2020-07-25

**Authors:** Shulong Zhang, Qi Han, Kaihua Zhu, Quan Wang

**Affiliations:** ^1^ Department of General Surgery Xuhui District Central Hospital of Shanghai Shanghai China

**Keywords:** colorectal cancer, MiR‐27a, polymorphism, risk

## Abstract

**Background:**

Besides environment and living habits, such as a sedentary lifestyle, smoking and drinking, genetic variation also plays an important role in the development of colorectal cancer (CRC). This study was aimed to investigate the role of miR‑27a rs895819 polymorphism on CRC risk in Chinese population.

**Methods:**

In a case‐control study including 208 CRC and 312 age‐ and gender‐matched healthy control subjects, the rs895819 polymorphism was genotyped using the TaqMan allelic discrimination assay. Furthermore, a pooled analysis based on eligible studies was performed by using the STATA software.

**Results:**

Logistic regression analysis showed that the rs895819 polymorphism was not associated with CRC risk. However, a pooled analysis based on five studies from Chinese population showed a statistically significant association between the rs895819 polymorphism and CRC risk (GG vs AA: OR = 1.56, 95% CI = 1.27‐1.92, *P*z < .01; (AG + GG) vs AA: OR = 1.14, 95% CI = 1.01‐1.30, *P*z = .04; GG vs (AG + AA): OR = 1.54, 95% CI = 1.27‐1.88, *P*z < .01; G vs A: OR = 1.20, 95% CI = 1.09‐1.33, *P*z < .01).

**Conclusion:**

Our study suggests that miR‑27a rs895819 polymorphism plays an important role in CRC risk in Chinese population and may serve as a valuable biomarker for predicting an individual's susceptibility to CRC.

## Graphical abstract

Rs895819 polymorphism located in miR‐27a was associated with colorectal cancer risk in Chinese population.

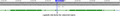

## 1 INTRODUCTION

MicroRNAs (miRNAs) are a class of small non‐coding RNAs participating in the regulation of the expression of downstream genes by targeting the 3′‐untranslated region (3′‐UTR) of the corresponding mRNAs. Growing evidence suggests that miRNAs play a major role in the development of cancer.^1^ For instance, miR‑27a, located on chromosome 19, has been demonstrated to be abnormally expressed in many types of cancer, including colorectal cancer (CRC).^2^, ^3^, ^4^ Gao et al found that miR‑27a highly expressed in patients with colon cancer could enhance proliferation and invasion of colon cancer cells.^4^ Liu et al^5^ found that miR‑27a could promote proliferation, migration, and invasion of CRC by targeting FAM172A and might serve as a diagnostic and prognostic biomarker of CRC. These findings indicated that miR‑27a could function as an oncogene in CRC.

Besides environment and living habits, such as a sedentary lifestyle, smoking, and drinking, genetic variation also plays an important role in the development of CRC.^6^, ^7^ Rs895819 polymorphism is a single nucleotide polymorphism (SNP) located in the terminal loop of pre‐miR‐27a and has been reported to be associated with the risk of cancer, such as non‐small cell lung cancer, cervical cancer, diffuse large B‐cell lymphoma, and CRC.^8^, ^9^, ^10^, ^11^ Although several studies have suggested that the rs895819 polymorphism was associated with CRC risk in Chinese population, these results need to be further confirmed.^11^, ^12^, ^13^ Hence, we conducted a case‐control study and pooled analysis to investigate the effect of the rs895819 polymorphism on CRC risk in Chinese population.

## 2 MATERIALS AND METHODS

### 2.1 A case‐control study

A total of 208 CRC patients and 312 healthy check‐up individuals matched with age and gender were recruited from Xuhui District Central Hospital of Shanghai between October 2017 and December 2019. All enrolled individuals were Chinese Han population. The mean age (mean ± SD) of CRC patients and healthy individuals was 60.1 ± 8.6 and 59.8 ± 8.1 years, respectively (*P* = .69). The ratio of male and female was 156:52 and 230:82 in CRC patients and healthy individuals, respectively (*P* = .74). CRC patients were diagnosed by histological examination. Written informed consent was obtained from all participants. The research protocol was approved by the ethics committee of Xuhui District Central Hospital of Shanghai.

Genomic DNA was extracted from peripheral blood of all participants by the QIAamp DNA mini Kit (Qiagen, Hilden, Germany) according to the manufacturer's instruction. The miR‐27a rs895819 polymorphism was detected using the TaqMan allelic discrimination assay. The primer and probe sequences and reaction conditions were used according to a description of previous literature.^14^ In addition, 10% samples were randomly selected to DNA sequencing. The results showed 100% concordance.

### 2.2 A pooled analysis

**FIGURE 1:**
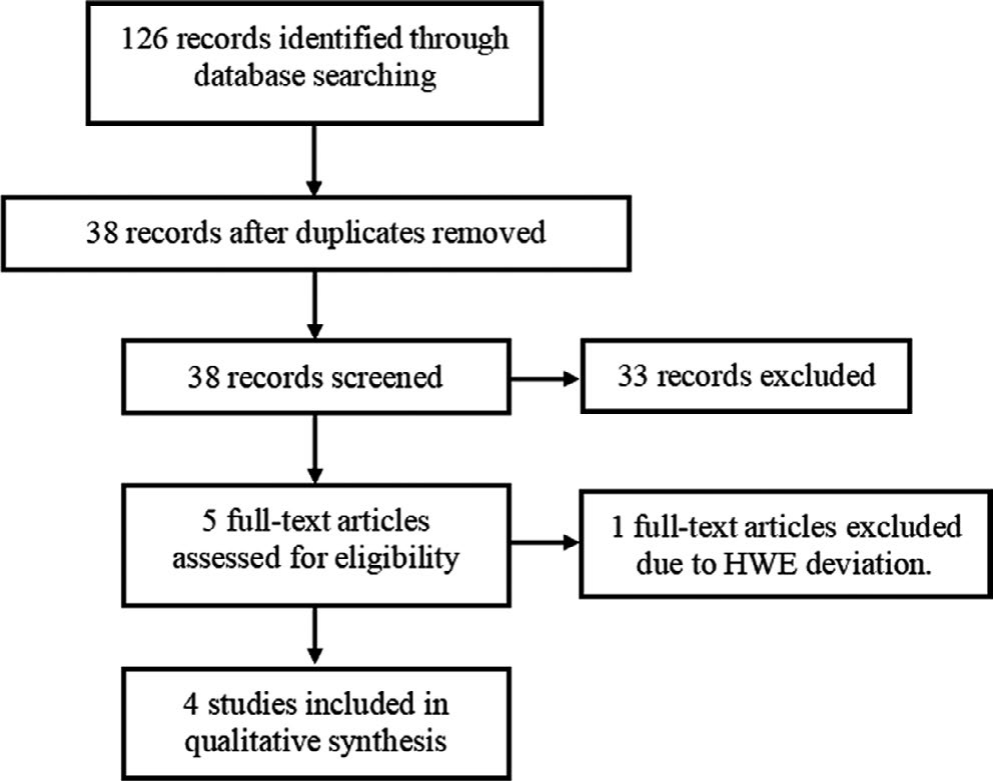
Flowchart of study selection

PubMed, Embase, and CNKI databases were independently retrieved by two investigators to obtain eligible literatures on the association of rs895819 polymorphism with CRC risk in Chinese population. Flowchart of study selection was shown in Figure 1. The following search terms were used: “MIR27A or miR‐27a”, “rs895819 or polymorphism or variant” and “colorectal cancer or CRC”. Last retrieval date was on April 2, 2020. Eligible literatures should meet all the following criteria: (a) case‐control design for assessing the association of the rs895819 polymorphism with CRC risk among Chinese population; (b) detailed genotype data for calculating pooled odds ratio (OR) and 95% confidence interval (CI); (c) *P* value for Hardy‐Weinberg equilibrium (*P*_HWE_) in the control group > .05. The relevant data, including the first author's name, publication year, country, cancer type, genotyping method, and genotype frequency distribution of the rs895819 polymorphism in cases and controls, were extracted independently by two investigators (Table 1). In addition, two investigators independently assessed the quality of each qualified study using the quality assessment criteria.^15^, ^16^ Quality scores of each study ranged from 0 to 15. Studies with scores ≤9 were categorized into low quality, while those with scores >9 were considered as high quality. Any discrepancies would be resolved by discussion.

**TABLE 1:** The relevant data from eligible literatures

Author	Year	Country	Cancer	Genotyping	Cases				Controls				*P*_HWE_
					AA	AG	GG	Total	AA	AG	GG	Total	
Jiang et al	2016	China	CRC	TaqMan	245	176	87	508	275	222	65	562	.05
Bian et al	2015	China	CRC	TaqMan	199	143	70	412	205	166	41	412	.39
Cao et al	2014	China	CRC	PCR‐RFLP	92	113	49	254	114	93	31	238	.09
Zhang	2012	China	CRC	PCR‐RFLP	248	178	37	463	270	166	32	468	.35

### 2.3 Statistical analysis

In the case‐control study, chi‐square test and Student's *t* test were used to compare the gender and age distribution between cases and controls, respectively. *P*_HWE_ in the control group was calculated by a goodness‐of‐fit chi‐square test. Logistic regression was used to assess the association of rs895819 polymorphism with CRC risk, which was adjusted for age and gender. The above analyses were performed using the SAS software, version 8.0. (SAS Institute, Cary, CA, USA).

In the pooled analysis, the pooled ORs with 95% CIs were calculated based on the random or fixed effect model under the additive (G vs A), dominant [(GG + GA) vs AA], recessive [GG vs (GA + AA)], and codominant model (GG vs AA; GA vs AA). Between‐study heterogeneity was evaluated through the chi‐square‐based Q test and *I* ^2^ index. If a significant Q‐statistic (*P*_het_ < .05) or *I* ^2^ > 50% indicating the existence of between‐study heterogeneity, the random effect model was used. Otherwise, the fixed effect model was used. In addition, sensitivity analysis was performed by omitting each study in turn to assess the consistency of the results. Begg's and Egger's tests were used to evaluate potential publication bias. *P* < .05 was set as the criterion of statistical significance. These analyses were conducted using the Stata software, version 12.0 (StataCorp, College Station, Texas). Furthermore, false‐positive report probability (FPRP) was calculated with threshold value = .2, prior probability = .01 and OR value = 1.50 to evaluate the significant findings.^17^ The significant result with an FPRP value < .2 was considered a noteworthy finding.

## 3 RESULTS

**FIGURE 2:**
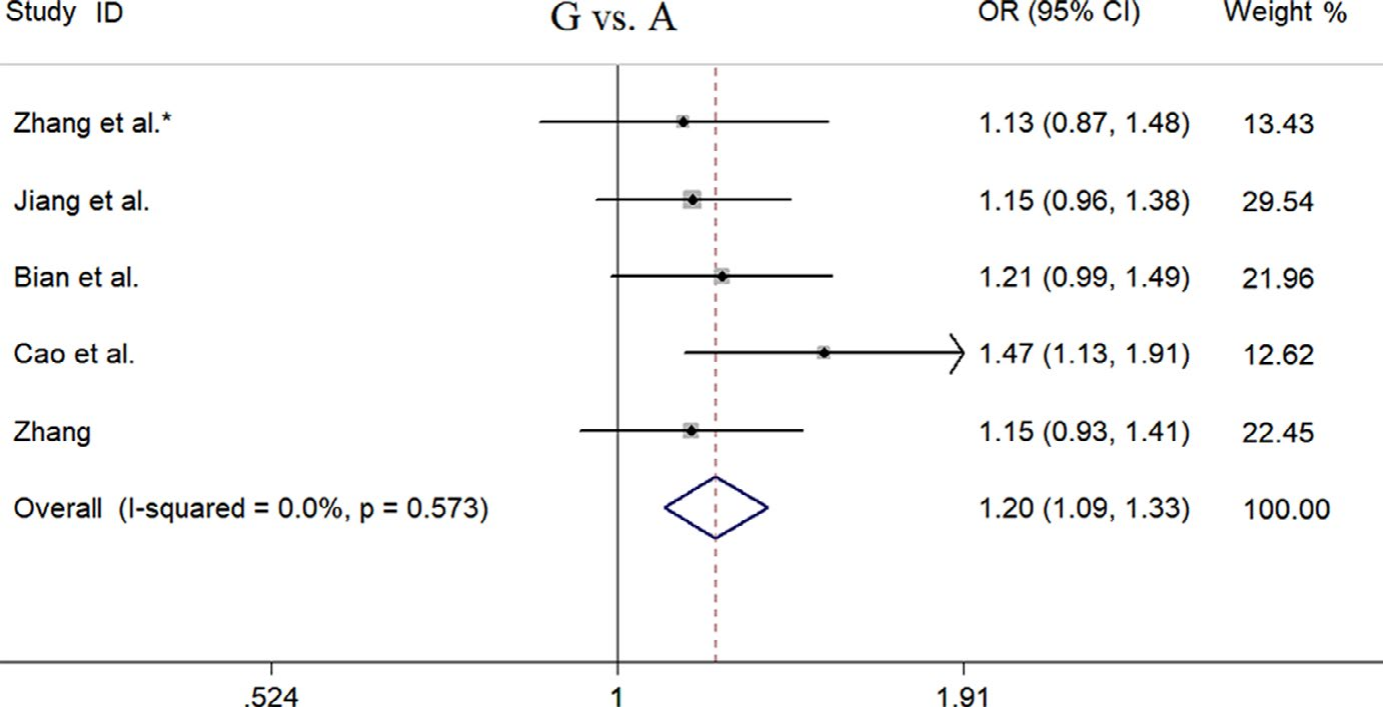
Forest plot of meta‐analysis on miR‐27a rs895819 polymorphism and colorectal cancer risk under G vs A

In the current case‐control study, there were no significant differences in the distribution of age or gender between cases and controls. Genotype distributions had no deviation from HWE in control groups (*P*_HWE_ = .75). Logistic regression analysis showed that rs895819 polymorphism was not significantly associated with CRC risk in a Chinese population (Table 2). However, a pooled analysis based on five studies with 1845 CRC patients and 1992 healthy individuals showed that rs895819 polymorphism was significantly associated with CRC risk in Chinese population (Table 3). Under the codominant model, we found that compared with the reference genotype (AA), the GG genotype of rs895819 was significantly associated with an increased risk of CRC (OR = 1.56, 95% CI = 1.27‐1.92, *P*z < .01). Under the dominant [(AG + GG) vs AA: OR = 1.14, 95% CI = 1.01‐1.30, *P*z = .04], recessive [GG vs (AG + AA): OR = 1.54, 95% CI = 1.27‐1.88, *P*z < .01], and additive [G vs A: OR = 1.20, 95% CI = 1.09‐1.33, *P*z < .01] models, significant associations between rs895819 polymorphism and CRC risk were also observed (Table 3 and Figure 2). Under the additive (G vs A), recessive [GG vs (GA + AA)], codominant model (GG vs AA), FPRP values were .048, .005 and .007, respectively. Furthermore, the results of both Begg's test and Egger's test did not show publication bias in the pooled analysis (Table 4). Sensitivity analysis showed that the pooled OR was not significantly changed by omitting each study at a time under the additive, recessive, or codominant model. However, under the dominant model, the pooled OR was significantly changed after removing Cao's or Zhang's study.

**TABLE 2:** A case‐control study of association between miR‑27a rs895819 polymorphism and CRC risk

Comparison model	Genotype and allele	Case (%) (N = 208)	Controls (%) (N = 312)	OR (95% CI)	*P* value
Codominant model	AA	100 (48.1)	157 (50.3)	Reference	
	GA	83 (39.9)	127 (40.7)	1.02 (0.70‐1.48)	.92
	GG	25 (12.0)	28 (9.0)	1.39 (0.77‐2.53)	.27
Dominant model	AA	100 (48.1)	157 (50.3)	Reference	
	GA + GG	108 (51.9)	155 (49.7)	1.09 (0.77‐1.54)	.64
Recessive model	AA + GA	183 (88.0)	284 (91.0)	Reference	
	GG	25 (12.0)	28 (9.0)	1.38 (0.78‐2.44)	.27
Additive model	A	283 (68.0)	441 (70.7)	Reference	
	G	133 (32.0)	183 (29.3)	1.13 (0.86‐1.47)	.38

**TABLE 3:** Pooled analysis of the association between miR‑27a rs895819 polymorphism and CRC risk

Comparison	Heterogeneity		Effect model	OR (95% CI)	*P*_Z_
	*P*_het_	*I* ^2^			
(AG + GG) vs AA	.32	15.5%	Fixed	1.14 (1.01, 1.30)	.04
GG vs (AG + AA)	.73	0.0%	Fixed	1.54 (1.27, 1.88)	<.01
GG vs AA	.76	0.0%	Fixed	1.56 (1.27, 1.92)	<.01
AG vs AA	.16	39.7%	Fixed	1.04 (0.90, 1.19)	.60
G vs A	.57	0.0%	Fixed	1.20 (1.09, 1.33)	<.01

**TABLE 4:** Assessment of publication bias in the pooled analysis

Comparison	*P* value of Begg's test	*P* value of Egger's test
(AG + GG) vs AA	.22	.23
GG vs (AG + AA)	.22	.39
GG vs AA	1.00	.94
AG vs AA	.22	.32
G vs A	.46	.40

## 4 DISCUSSION

In recent years, the role of miR‑27a rs895819 polymorphism on CRC risk has been investigated in Chinese population.^11^, ^12^, ^13^, ^18^, ^19^ Bian et al found that GG genotype of the rs895819 polymorphism was positively associated with an increased risk of CRC in codominant (GG vs AA) and recessive (GG vs AA/GA) models, indicating that GG genotype of the locus might increase CRC risk in Chinese population.^11^ Jiang et al found that GG genotype of the rs895819 polymorphism was associated with an increased risk of CRC in Chinese population, and individuals with A allele (AA/AG) were significantly associated with a decreased risk for CRC.^12^ Cao et al^13^ observed that subjects with variant genotypes (AG + GG) of the rs895819 polymorphism had a significantly increased risk of CRC compared with subjects with AA genotype. Wang et al found that the GG genotype of the rs895819 polymorphism was significantly associated with CRC risk when taking the AA genotype as a reference.^18^ Zhang's study found that rs895819 polymorphism was associated with CRC risk in Chinese female population, and compared with female individuals with AA genotype, female individuals with AG genotype had a significantly increased risk of CRC.^19^ In order to further confirm the association of rs895819 polymorphism with CRC risk in Chinese population, a case‐control study with 208 CRC patients and 312 healthy individuals was carried out. The result showed that rs895819 polymorphism was not significantly associated with CRC risk in Chinese population, which was not consistent with the result of previous studies. The inconsistent result might be due to a small sample size or lack of adjustment for lifestyle and environmental factors. Subsequently, we obtained four relevant studies from several electronic databases according to inclusion criteria. The four studies from Chinese population contained 1637 CRC patients and 1680 healthy individuals. Pooled analysis based on current case‐control study and previous four studies showed that rs895819 polymorphism was significantly associated with CRC risk in Chinese population, which was consistent with those of a previous meta‐analysis.^20^ Genotypes GG and allele G were significantly associated with an increased risk of CRC.

Although the pooled analysis revealed the role of miR‑27a rs895819 polymorphism on CRC risk in Chinese population, some limitations should be addressed. Firstly, the pooled result was not adjusted for potential confounding factors, while a more precise assessment should be performed if individual information, such as smoking, drinking, and red meat intake, was available. Furthermore, sensitivity analysis showed that the pooled OR was unstable under the dominant model, suggesting that the result needed to be further confirmed by more case‐control studies.

## 5 CONCLUSION

Our study observed a significant association between miR‑27a rs895819 polymorphism and CRC risk in Chinese population. The rs895819 polymorphism might serve as a valuable biomarker for predicting an individual's susceptibility to CRC.

## AUTHOR CONTRIBUTIONS

Shulong Zhang, Qi Han, Kaihua Zhu, and Quan Wang conceived of the study and participated in its design. Shulong Zhang, Qi Han, and Kaihua Zhu conducted the systematic literature review. Shulong Zhang and Quan Wang performed the experiment and data analyses. Shulong Zhang drafted the study.
